# Fungal allergy in asthma–state of the art and research needs

**DOI:** 10.1186/2045-7022-4-14

**Published:** 2014-04-15

**Authors:** David W Denning, Catherine Pashley, Domink Hartl, Andrew Wardlaw, Cendrine Godet, Stefano Del Giacco, Laurence Delhaes, Svetlana Sergejeva

**Affiliations:** 1 The National Aspergillosis Centre, University Hospital of South Manchester, The University of Manchester, Manchester Academic Health Science Centre, Manchester, UK; 2 Education and Research Centre, UHSM, Southmoor Road, Manchester M23 9LT, UK; 3 Leicester Institute for Lung Health and Respiratory Biomedical Research Unit, Department of Infection Immunity and Inflammation, University of Leicester, Glenfield Hospital, Groby Road, Leicester LE3 9QP, UK; 4 Department of Pediatrics, Infectious Diseases & Immunology, University of Tübingen, Tübingen, Germany; 5 Department of Infectious Diseases, CHU la Milétrie, Poitiers, France; 6 Department of Medical Sciences “M. Aresu”, University of Cagliari, Cagliari, Italy; 7 Biology & Diversity of Emerging Eukaryotic Pathogens (BDEEP), Center for Infection and Immunity of Lille (CIIL), INSERM U1019, CNRS UMR8204, IFR142, Lille Pasteur Institute, Lille Nord de France University (EA4547), Lille, France; 8 Department of Parasitology–Mycology, Regional Hospital Center, Faculty of Medicine, Lille, France; 9 Translational Immunology Group, Institute of Technology, Tartu University, Tartu, Estonia; 10 North Estonia Medical Centre, Tallinn, Estonia

**Keywords:** Aspergillus, Severe asthma, SAFS, ABPA, ABPM, Corticosteroid, Eosinophil, IgE, Itraconazole, Hypertonic saline

## Abstract

Sensitization to fungi and long term or uncontrolled fungal infection are associated with poor control of asthma, the likelihood of more severe disease and complications such as bronchiectasis and chronic pulmonary aspergillosis. Modelling suggests that >6.5 million people have severe asthma with fungal sensitizations (SAFS), up to 50% of adult asthmatics attending secondary care have fungal sensitization, and an estimated 4.8 million adults have allergic bronchopulmonary aspergillosis (ABPA). There is much uncertainty about which fungi and fungal allergens are relevant to asthma, the natural history of sensitisation to fungi, if there is an exposure response relationship for fungal allergy, and the pathogenesis and frequency of exacerbations and complications. Genetic associations have been described but only weakly linked to phenotypes. The evidence base for most management strategies in ABPA, SAFS and related conditions is weak. Yet straightforward clinical practice guidelines for management are required. The role of environmental monitoring and optimal means of controlling disease to prevent disability and complications are not yet clear. In this paper we set out the key evidence supporting the role of fungal exposure, sensitisation and infection in asthmatics, what is understood about pathogenesis and natural history and identify the numerous areas for research studies.

## Introduction

Fungal exposure is a daily fact of human existence, which infrequently results in disease. Yet fungal allergy drives asthma severity in very large numbers of people affected by severe asthma. Available statements from different medical associations are unequivocal in declaring that fungi are sensitizers and exacerbate allergic asthma (American College of Occupational and Environmental Medicine [1], Institute of Medicine, American Academy of Allergy, Asthma and Clinical Immunology [2] and American College of Medical Toxicology). Increasing rates of fungi-associated occupational asthma are also of concern. In contrast to allergy to other environmental agents, antifungal therapy is available, yet our knowledge of who, when and how to treat is in its infancy. As with other allergens, immunization could be helpful but has barely been addressed.

The European Academy of Allergy and Clinical Immunology (EAACI) Asthma Section decided to address this deficiency by forming a Task Force to address ‘Fungal Allergy in Asthma’. The first product of the Task Force is a summary of what is and what is not known about this topic, written from the perspective of the progressive practicing clinician. It is focused on the lower respiratory tract, does not address cystic fibrosis (CF) in any depth and highlights research needs in the area. Other consensus reviews and statements relevant to this topic have described the status of allergic bronchopulmonary aspergillosis (ABPA) in cystic fibrosis [3], lower airways interaction with fungi and its clinical consequences [4] and the whole topic of ABPA [5], the last with recommendations for a new definition of ABPA.

### Conceptual background

While fungal exposure is universal, sensitisation and disease are not. Very early life airborne contact with fungi is well demonstrated by studies with *Pneumocystis* serology and pneumonia in healthy children and those with cancer [6]. Broadly speaking, fungi can cause problems to the lung in two ways; either by acting as aeroallergens or as a pathogen causing infection. Some fungi can do both, often simultaneously. To cause infection in the lung the fungus has to be able to grow at body temperature and this property is restricted to a relatively narrow range of fungi, particularly yeasts and members of the *Aspergillus* and *Penicillium* genera. The commonest fungus causing lung infections is *Aspergillus fumigatus*, although other *Aspergillus* spp. are also implicated [7]. Fungal allergens, which can cause rhinitis and asthma, but rarely cause infection, include spores from the plant pathogens *Cladosporium* and *Alternaria* spp. A third potential cause of ill-health from fungi are volatile organic compounds and mycotoxins released by moulds such as *Stachybotrys* spp*,* which remains controversial and will not be discussed here in depth.

#### Sensitisation and allergy

*Allergy* is an inflammatory response caused by an environmentally delivered and often non-pathogenic agent and is caused by an exaggerated immune response rather than the pathogenic, pharmacological or toxic properties of the primary agent. As fungi are complex eukaryotes, all forms of allergic immune response [8] should be considered as potentially leading to fungal allergy, although the most well-recognised clinical responses, such as asthma and rhinitis caused by *Alternaria alternata*, are mediated in a straightforward immunoglobulin (Ig) E/TH_2_ manner. The stipulation on including evidence of an inflammatory response in the definition of allergic disease is to distinguish *allergy* from *sensitisation*. Many people with elevated specific serum (s)IgE (or for that matter other intermediates of immune response) against a certain agent (sensitisation) do not develop symptoms when exposed to that agent. However, this is not a fixed difference as sensitisation can evelove into allergy depending on the level of exposure, co-factors present at the time of exposure and the age of person, with periods in their life when they develop symptoms and periods when they have sub-clinical disease or are in complete remission induced by immune tolerance.

#### Infection

Viable microorganisms including fungi can have a range of interactions with their human host. *Infection* refers to the presence of a microorganism, which leads directly to ill-health as a result of its pathogenic properties. *Colonisation* refers to any situation where a microorganism becomes established in a new environment and doesn’t imply any particular relationship between the organism and the host. Both pathogenic and non-pathogenic organisms can act as commensals. The ability of microorganisms cause disease depends on their pathogenic potential, the number of organisms, the integrity of host defense, the strain of the organism and no doubt other factors, which are yet to be defined. If there is evidence of tissue dysfunction which is likely to be due to the presence of the microorganism then the term *infection* is preferable to colonisation.

**Figure 1:**
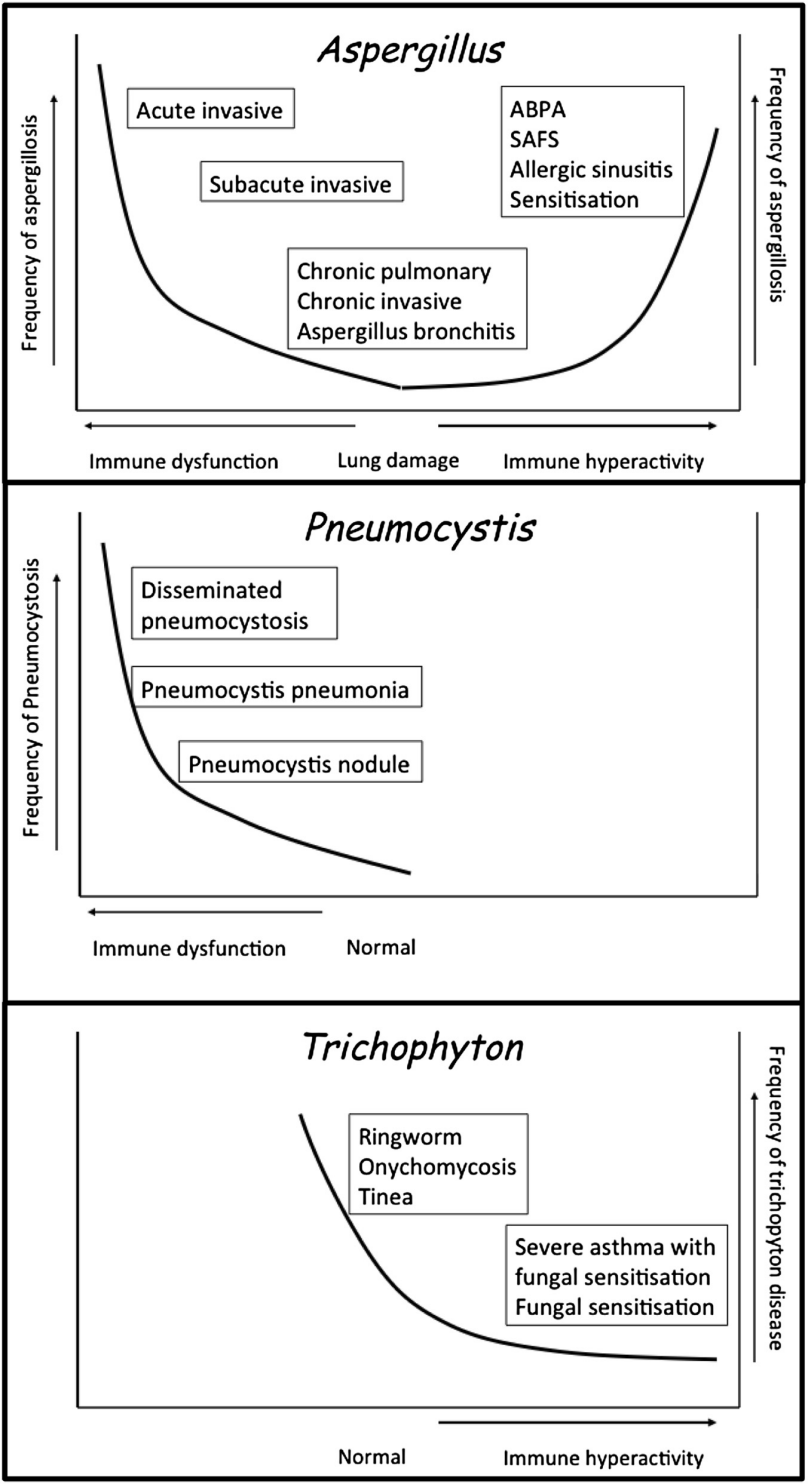
**Patterns of fungal interactions with humans, illustrating different host pathogen interactions, based on the host damage response framework**[9]**.**

Fungi can cause a number of different types of infection, separately from their ability to act as sensitisers. Contrasting patterns of fungi-host interactions are illustrated in Figure 1, with *A. fumigatus* remarkable as it can cause invasive infection in the immunocompromised, chronic pulmonary aspergillosis and *Aspergillus* bronchitis in non-immunocompromised individuals with underlying lung damage, and allergic disease of the upper and lower airways. In contrast, *Pneumocystis* is a pathogen of the immunocompromised, but not an allergenic fungus or sensitizer, which greatly differs from the primary skin pathogen *Trichopyton interdigitale* (and other species) which causes cutaneous infection in hundreds of millions of people, cannot grow in the lung, but is a common sensitising fungus associated with severe asthma.

#### Asthma

This position paper concerns fungal allergy in *asthma*. However asthma itself is not a straightforward term and requires clarification. Asthma can be defined in two ways, descriptively and rather loosely as a multifactorial chronic inflammatory disease of the airways with an associated set of typical symptoms and evidence of variable airflow obstruction, or more strictly as a physiological abnormality in which there is documented rapid changes in the resistance to airflow and increased sensitivity of the airway smooth muscle to bronchoconstricting stimuli (airway hyperresponsiveness (AHR)) [10]. Bio-statistical approaches to measuring heterogeneity in asthma have suggested that asthma as broadly defined is comprised of a number of distinct endotypes, not all of which have obvious AHR or variable airflow obstruction [11,12], and improvement in symptoms of asthma are not always reflected in better physiological measurement of airflow. Many conditions can mimic asthma, or occur alongside it, confusing the clinical picture.

### Current disease concepts, definitions and terminology

**Figure 2:**
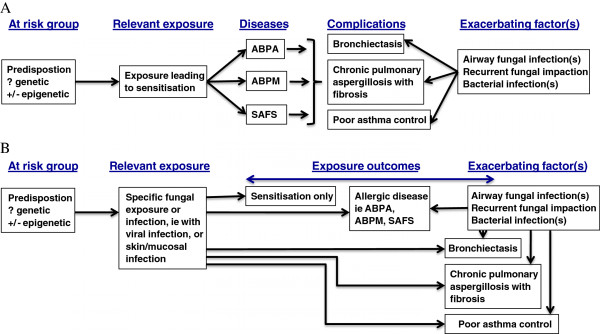
**Two contrasting disease models (A and B), with common elements of risk and exposure, but different outcomes.** Model **A** illustrates a linear relationship between disease and complications, whereas model **B** provides independent consequences of fungal exposure, which may or may not be associated. In both models, it is proposed that airway colonization/infection with fungus, and possibly fungal impaction from breathing airborne non-pathogenic fungi, and/or bacterial infection, continue to drive the inflammatory process.

We have tabulated the current and proposed definitions of fungal allergy, sensitisation and related conditions in Table 1. ABPA was first described in 1952 [13], and the criteria for diagnosis were defined during the 1970s and 1980s [14-17]. It is recognised that occasionally ABPA can occur in the absence of asthma or CF, with other criteria positive. The cutoff for total serum IgE has not been rigorously addressed in asthma, although in CF in the UK a cutoff of >180 IU/mL was 91% sensitive and 90% specific [18] in contrast to an Indian cohort of asthmatic patients with ABPA in whom a total serum IgE of 2346 IU/mL (sensitivity 87.5%, specificity 66.9%) appeared optimal [5]. The utility of the Patterson criteria have been prospectively evaluated and compared with recently introduced criteria by Agarwal [19]. Use of 6 (but not 5, 7 or 8) ‘Patterson criteria’ performed best in a cohort of 372 asthmatics in northern India [19]; 98% of the 56 patients with ABPA identified in this study had bronchiectasis, potentially affecting the performance of the definition. In addition, some clinicians use expectoration of brown coloured mucus plugs as a minor diagnostic criteria for ABPA [20], observed in 31 to 69% of patients [5]. These results raise questions about the optimal disease model and as a consequence the diagnostic criteria for ABPA (see Figure 2).

**Table 1:** Definitions

**Entity**	**Current definition**	**Proposed definition**	**Comments**
Fungal allergy	Immune-mediated inflammatory response to a fungus sometimes leading to tissue damage	Same, being inclusive of all allergic immunopathologies	Demonstrating and documenting ‘Tissue damage’ can sometimes be difficult.
Fungal sensitisation	Immune-mediated response to a fungus, without evidence of inflammation or tissue damage, usually documented by an elevated fungal-specific IgE.	Same	Tends to reflect specific-IgE response (or skin prick test result) only.
Fungal colonisation	None	1. One (or preferably two or more) respiratory sample (s) positive for a fungus by culture or PCR	Such criteria may apply to other filamentous fungi, but not Candida. They need to be tested in prospective studies.
		2. No new major respiratory symptoms	
		3. No evidence of ABPA or other forms of aspergillosis	
		4. No overt immunocompromise	
		5. Negative fungal specific IgG in serum.	
ABPA	1. Asthma (or CF) with deterioration of lung function	1. Asthma (or CF)	No definition addressed robustly with prospective study and combinations of diagnostic criteria.
	2. Elevated total serum IgE of >1000 ng/ml (>417 IU/ml)	2. *A. fumigatus* skin test positive or elevated *A. fumigatus* IgE levels	
	3. Elevated *A. fumigatus* specific IgE and/or IgG antibodies	3. Elevated total serum IgE of >1000 IU/ml	
		+ 2 of the following:	
		a. Positive *A. fumigatus* IgG or precipitating antibodies	
		b. Radiographic opacities consistent with ABPA	
		c. Eosinophil count >500 cells/uL	
	4. Immediate *Aspergillus* species skin test reactivity		
	5. Eosinophilia (>1,000/uL)		
	6. Presence of central (or proximal) bronchiectasis		
	7. Chest radiographic infiltrates		
	8. High attenuation mucus present.		
Allergic bronchopulmonary mycosis (ABPM)	1. Asthma (or CF) with deterioration of lung function	No new proposal, but similar to ABPA, with substitution of a different fungal specific tests.	Too rare to develop patient cohorts to formally validate a definition.
	2. Elevated total serum IgE of >1000 ng/ml (>417 IU/ml)		
	3. Elevated fungal specific IgE and/or IgG antibodies		
	4. Immediate fungal species skin test reactivity		
	5. Eosinophilia (>1,000/uL)		
	6. Presence of central (or proximal) bronchiectasis		
	7. Chest radiographic infiltrates		
Severe asthma with fungal sensitisation (SAFS)	1. Severe asthma		Severe asthma is a variable, usually treatment-based entity.
	2. Total IgE <1,000 IU/mL		Variable performance of different skin and sIgE test reagents, makes SAFS an imprecise entity until diagnostics improve.
	3. Sensitisation to any fungus by skin prick test or sIgE		
Aspergillus bronchitis	None	1. Multiple respiratory sample positive for *Aspergillus* spp by culture or PCR	Few patients reported. Some cases are caused by non-*fumigatus* species, and so serology criterion may be falsely negative.
		2. Major respiratory symptoms for >4 weeks	
		3. No evidence of ABPA or other forms of aspergillosis	
		4. No overt immunocompromise	
		5. Positive *Aspergillus* IgG or precipitins in serum.	

Fungal allergy, fungal sensitisation and fungal colonisation may be clinically silent.

There are some other difficulties with current diagnostic criteria. One of these is overlapping syndromes: separation of ABPA from Hyper IgE Syndrome (Job’s syndrome) [21], separation of ABPA complicated by bronchiectasis and *Aspergillus* bronchitis [22], separation of ABPA from chronic pulmonary aspergillosis with a prominent TH_2_ response, including raised serum total and sIgE [23], and Churg-Strauss syndrome in the absence of biopsy as common examples. While the presence of central (or proximal) bronchiectasis and/or chest radiographic infiltrates are usually cited as primary criteria of ABPA [4,24], a recent consensus group suggested that bronchiectasis should be regarded as a complication of ABPA and avoidable may be with earlier therapy [5].

It has been recognised that other fungi can induce a disease process similar to ABPA, normally called allergic bronchopulmonary mycosis (ABPM) [25]. Many different fungi can be implicated and the remarkable feature in all is a very high total and specific serum IgE to one or more non-*Aspergillus* fungi, and other findings usually found in ABPA. *Candida albicans* is the most commonly reported associated fungus with *Bipolaris* species, *Schizophyllum commune* and *Curvularia* species the next most common.

In 2006, the term severe asthma with fungal sensitisation (SAFS) was introduced [26] and subsequently shown to be responsive to antifungal therapy [27]. For consistency, the definition of severe asthma should be uniform, but is often not fully distinguished from dysfunctional breathing and tracheomalacia. In addition, current definitions of severe asthma generally reflect treatment intensity; the WHO identified 3 patterns: 1) untreated severe asthma, due to undiagnosed asthma or unavailability of therapy, 2) difficult-to-treat severe asthma (due to adherence issues, inappropriate or incorrect use of medicines, environmental triggers or co-morbidity), and 3) treatment-resistant severe asthma, including asthma for which control is not achieved despite the highest level of recommended treatment or asthma which is controlled only with the highest level of recommended treatment [28]. Asthma severity may also change with antifungal therapy, which was vividly illustrated by a report of antifungal therapy allowing one third of patients having their asthma severity downgraded [29]. The definition of SAFS includes people sensitized to purely allergenic fungi such as *Alternaria alternata* but sensitisation to thermotolerant fungi is not restricted to people with severe asthma, nor indeed to people with asthma, creating uncertainty with respect to the best disease model. Sensitization to *A. fumigatus* is more common in severe asthma but it is not uncommon to find patients who present with severe fixed airflow obstruction on a background of apparently mild to moderate asthma who are sensitized to *A. fumigatus*, suggesting the tissue damage associated with allergy to *A. fumigatus* is not restricted to severe disease. While ABPA was accepted as an endotype of asthma by another EAACI Task Force [12], SAFS was not and remains a pragmatic definition to enable an antifungal therapy trial. This issue needs resolution, possibly by genotyping patients with different forms of asthma [30], if genotypes allow such classifications, which they may not.

Fungal sensitisation is also found in allergic rhinosinusitis, without asthma or ABPA, and is a diagnostic criterion for allergic *Aspergillus* rhinosinusitis [31,32]. Recently over 10% of patients with chronic obstructive airways disease (COPD) were found to be sensitized to *A. fumigatus* and this was associated with worse pulmonary function [33].

The role of airway colonization or infection by fungi, is probably significant, and is most notably related to *A. fumigatus*. About 60% of asthmatics sensitised to *A. fumigatus* have *A. fumigatus* growing in their sputum on a single sample (compared to ~5% of normal subjects) and this increases to 80% if multiple samples are obtained over time (AJW/CHP personal observation). Importantly, these patients have impaired lung function and increased rates of bronchiectasis compared to matched controls suggesting evidence of tissue damage [24]. Likewise those asthmatics sensitised to *A. fumigatus* have a much higher rate of bronchiectasis [34], and children sensitised to *Alternaria* are more likely to have persistent asthma in adulthood [35]. Our supposition is that fungal airway infection induces an ongoing allergic stimulus, as although most fungal conidia (spores) release some allergen(s), many more are produced after spore swelling and germination [36]; before germination the conidia are covered in hydrophobin proteins, which elicit no immune reaction [37]. Preformed allergen from hyphal fragments may elicit an immune response, even in the absence of local germination and growth [38], possibly explaining why increased environmental exposure to *Cladosporium* spp. or *Alternaria alternata*, for example are associated with worse asthma.

Another clinical entity, widely acknowledged in several southern countries, is “*Tricophyton Asthma*”. There are multiple lines of evidence for sensitisation to *Tricophyton* proteins in asthma patients [39-42], where inhalation and/or dermal absorption of *Trichophyton* antigens are currently considered as possible routes of exposure [39,41]. Another study showed a high positive skin test rate in asthmatic patients with *Trichophyton* infection, confirming that the presence of cutaneous fungal infection is a crucial determinant of sensitivity to *Trichophyton*[42], regardless of the patients’ atopic status [41]. A Japanese study showed that specific IgE response to *Trichophyton* doubles to 32.4% with increasing severity of asthma, suggesting an independent determinant of asthma severity [40]. Otherwise, there are few data about the relative frequency of *Trichophyton* sensitisation in asthma. One recent UK study found a frequency of 17% in polysensitised severe asthmatics; 10% were sensitised to *Trichophyton* as a single mould sensitisation, but only 2.5% were sensitized to *Trichophyton* alone [43].

What terminology can we use to capture the full spectrum of disease characterized by fungal sensitization/allergy, often associated with fungal airway infection? ABPA/M has a well-established but restrictive definition, leaving out the majority of patients sensitized to *A. fumigatus* and related moulds without evidence of lung damage. Perhaps ABPA, ABPM and SAFS are simply the severe end of the same spectrum of disease. On the other hand, sensitization to moulds is also seen in people with mild asthma and good lung function or allergic rhinitis where the mould may simply act as an allergen, although it is possible that over time these people will develop fixed airflow obstruction, bronchiectasis and even chronic pulmonary aspergillosis and parenchymal fibrosis. Ideally, definitions and inclusion criteria should be based on outcomes, for example treatment response to antifungal agents or risk of developing progressive lung damage. However, there are virtually no robust data to guide this approach. One simple option would be to adopt an inclusive approach and relax the criteria for ABPA/M to describe anyone with airway disease, evidence of allergy to thermotolerant fungi (as defined by a positive serum sIgE or SPT) and lung damage (fixed airflow obstruction, tree in bud shadowing on CT scan, bronchiectasis of any description, or lung fibrosis). In this model, people with airway disease and sensitisation to thermotolerant fungi, but no evidence of lung damage, would be regarded as at risk of developing future lung damage and kept under appropriate surveillance. If this model were adopted it would need to be followed by large-scale trials of antifungal and/or immunomodulatory agents and large longitudinal cohort studies powered to relate the various immunological and clinical features of ABPA/M in its new definition to the risk of lung damage and the benefits of eradication of airway fungal infection.

### Disease concept research priorities

• *Large longitudinal observational studies of people with airway disease including asthma with different severity and sensitization to A. fumigatus and other fungi powered to determine the relationship between development or progression of lung or airway damage and the clinical and immunological criteria currently used to define ABPA/M, SAFS and sensitisation.*

• *Large studies of effective anti-fungal agents in people with airway diseases including asthma of different severity and sensitization to A. fumigatus and other fungi powered to determine the relationship between treatment outcomes, the course of disease and the clinical and immunological criteria currently used to define ABPA/M and SAFS*.

### Natural history and key findings

ABPA has a natural history that is both poorly characterized and difficult to predict, but better understood than other entities of fungal allergy in asthma. ABPA does not necessarily occur in the most severe cases of asthma. Sensitization to fungi is rare in childhood, although ABPA occurs occasionally in children [44-46], as does the recently described SAFS. Sensitisation to *Alternaria* in childhood (age 6 years) is associated with persistent asthma in early adulthood [35], but no other fungal allergens were tested. The natural history of fungal sensitisation in mild or moderate asthma is not currently described at all.

Patterson et al. described five non-sequential stages of ABPA (acute, remission, exacerbation, steroid-dependent asthma and fibrosis) based on clinical presentation, including recurrent and/or chronic persistent symptoms [47,48]. This staging, however, does not necessarily reflect the disease’s progression. Although the disease may remit temporarily, it is more often a progressive disorder with recurrent, infrequent acute episodes that cause successive bronchial damage. Tissue damage tends to be greatest in the proximal airway region, where mycelial plugs and antigen production are localised, giving rise to granulomatous airway inflammation that results in the characteristic proximal bronchiectasis [49]. In the early stages of ABPA, the bronchial wall is infiltrated with mononuclear cells and eosinophils. If mucoid impaction and atelectasis occur, they may be followed by bronchiolitis obliterans, granulomatous bronchiolitis, and pulmonary fibrosis [50].

A large variety of imaging changes are found in ABPA, and may be related to the phase of the disease. Lung infiltrates, bronchiectasis and mucoid impaction are the most common pathologic findings seen on a chest radiograph with ABPA. Transient changes include areas of consolidation. The pathologic basis of consolidation is unclear: it may be related to airway obstruction or eosinophilia. Although such areas of consolidation are usually transient, some patients present with permanent consolidation, perhaps due to endobronchial obstruction. When consolidation clears, it often leaves residual bronchiectasis. The pulmonary findings of ABPA on high-resolution CT (HRCT) include centrilobular nodules, bronchiectasis (often with mucoid impaction), fibrosis and cavitation. In SAFS, only bronchiectasis is well recognised, although both airway and peripheral pleuroparenchymal fibrosis, a manifestation of chronic pulmonary aspergillosis, is also described [51].

ABPA has been classified either in terms of ABPA-S and ABPA-CB (depending on, respectively, the absence (ABPA-S) or presence of bronchiectasis (ABPA-CB)) or in terms of ABPA-S (mild), ABPA-CB (moderate) and ABPA-CB-ORF (other radiologic findings) [52-54]. The prevalence of bronchial dilatation in patients with severe or persistent asthma ranges from 17% to 30%, compared with 90% or more in ABPA [55]. It has been thought that patients with ABPA-S represent the earliest stage of the disorder, but prolonged follow up of this group showed no interval development of bronchiectasis, despite exacerbations [56]. In a study of 126 patients, the clinical, spirometric and immunologic findings were not significantly different when classified in terms of either ABPA-S and ABPA-CB or ABPA-S, ABPA-CB and ABPA-CB-ORF [57].

High-attenuation mucus (HAM) is a characteristic radiologic finding seen in patients with ABPA [57], and not in any other asthma entity. A multivariate analysis showed that severity of bronchiectasis and presence of hyperattenuating mucus impaction are predictive of ABPA relapse, and that severity of bronchiectasis was an independent predictor of failure to achieve long term remission [58]. In the same study, HAM was shown to be present in 18% of patients diagnosed with ABPA. Reasons why hyperattenuating mucus is associated with poorer outcomes remain unclear; high attenuated mucus may be a more inspissated type of mucus. Perhaps this subgroup of patients has more severe inflammation and/or specific genetic alterations that favour the formation of HAM [58]. More research is therefore required to investigate the exact reason for this association. It is not clear whether bronchiectasis and pulmonary fibrosis constitute sequels of mucoid impaction in ABPA. Mucoid impaction as an entity is not well understood, and may be associated with *Aspergillus* bronchitis (usually complicating bronchiectasis) and obstructing bronchial aspergillosis (in immunocompromised patients).

The relapse frequency in ABPA is usually low, and reduced further by corticosteroid and antifungal therapy in many patients. Whether significant external fungal exposures precipitate relapse is not clear, but possible. Viral and bacterial respiratory infections may contribute to relapse, or to associated tissue damage and development of complications. It may be that remission of ABPA and SAFS occurs in older age, but this is not studied.

Pulmonary cavitation and fibrosis are often severe complications of ABPA, and may occur in SAFS. In one series, cavitation and atelectasis were shown to develop in, respectively, 20% and 46% of patients with ABPA [59]. Cavitation was observed to occur in patients with a chronic infiltrate and was thought to represent necrosis within an eosinophilic pneumonia. Cavitation may be reversible, following resolution of infection. The development of chronic pumonary aspergillosis (CPA) with or without cavitation may complicate ABPA and aspergillomas have been reported in up to 7% of patients with ABPA. Aspergilloma usually occurs in patients with longstanding ABPA and extensive lung destruction, even though this is not always the case: rapid formation of aspergilloma in early ABPA has also been described (before bronchiectasis)**,** implying that in some patients, it may be formed from direct parenchymal invasion [60]. In a recent study, ABPA was identified as an underlying condition in 18 (14.3%) and, more specifically, as the primary underlying condition in 15 (11.9%) out of 126 CPA cases, some with aspergillomas [51]. These findings support those from a study of aspergillomas where ABPA was identified as an underlying condition in 11.8% (10 out of 85) of cases reviewed [51]. The fact that ABPA is a relatively common underlying condition suggests that, it is not so much the presence of *Aspergillus* itself, as are the interaction of the lung’s condition (bronchiectasis/fibrosis) and corticosteroid exposure (which is the treatment of choice for ABPA) that are the risk factors for development of CPA. The radiological manifestations of early CPA in ABPA are not well delineated. Whether antifungal therapy arrests the progress of CPA complicating ABPA is not clear, but our anecdotal experience suggests it does. SAFS has also been associated with CPA, though infrequently [51].

Invasive aspergillosis complicating ABPA has also been rarely described, the first time in 1975 [61].

### Natural history research priorities

• *Large longitudinal observational studies of people with ABPA and SAFS, including detailed imaging, from as early a stage as possible after recognition to ascertain the relative frequency and associations with the different complications.*

• *Detailed study of mucoid impaction and hyper-attenuating mucus.*

• *Genetic and functional studies comparing patients with and without complications of ABPA and SAFS, including those with mild and severe bronchiectasis and those with early and advanced CPA.*

• *A better understainding of co-morbidities and their impact on natural history, is also required.*

### Epidemiology

#### Asthma prevalence and burden of fungal allergy in asthma

The Global Burden of Disease 2010 project estimated that asthma was ranked 43rd globally for years life lost [62], although 15th and 20th in Oceania and Eastern Europe respectively; 15th globally in terms of prevalence with an estimated 334 million people affected; 14th in terms of years lived with disability [63]; and 28th in terms of disability-adjusted life years [64]. An estimation of the adult burden of asthma using an alternative modeling approach based on the global initiative for asthma (GINA) yielded a global burden of 197 millions [65]. Utilising the 5 estimates of ABPA period prevalence (0.7-3.5%, median 2.5%) among referred patients to secondary care from South Africa, Ireland, Saudi Arabia, New Zealand and China there are 4.8 million adults with ABPA complicating asthma [65]. This estimate could be an overestimate because only a proportion of cases is referred to secondary care, or an underestimate because milder cases are not investigated in the community or referred, or were investigated previously and discharged back to the community.

Global or national SAFS prevalence estimates have not been made. The more severe asthma is, the higher is the frequency of *Aspergillus* and fungal sensitisation. Assuming that the worse 10% of adult asthmatics are severe and that fungal sensitisation has a minimum frequency of 33% in these people, about 6.5 million would be expected to have SAFS, although there is some crossover with ABPA patients who also have severe asthma [65]. It is not known how much regional or ethnic variation there is in SAFS prevalence.

#### Environmental exposure

Many recent studies describe mould-related respiratory effects in asthma [66-68]. Studies in environmental medicine have established a clear causal relationship in mould-induced asthma. Furthermore, these studies indicate that prevalence of adulthood asthma induced by moulds is increasing. The fraction of adulthood asthma incidence attributable to work is about 30% [69]. Adult-onset asthma is strongly associated with the level of fungal exposure [70]. Quite recently, moulds became the leading causative factor for occupational asthma (OA); notably in Finland. All *de novo* diagnosed cases of occupational asthma in Finland are obligatorily reported to the National Register, and by 2002 moulds become the most important causative agent (18% of cases in 1998-2002) [71]. Importantly, diagnosis of mould-induced asthma was confirmed by the allergen-specific challenge tests. The people affected are mostly white-collar employees, a population not generally considered as a risk group for OA. The same increasing trends have been shown in UK [72]. One of the most well established causes of occupational asthma associated with fungal allergens is alpha amylase from *Aspergillus oryzae* which is used as a additive in bakeries and can lead to ‘bakers asthma’ [73]. Alpha amylase has also been implicated in case reports in causing allergic reactions to white bread [74].

##### Outdoor

The data on the association between total fungal spore counts, spores grouped by their division or individual spore *taxa* and hospitalizations or visits to emergency departments due to asthma exacerbations are inconsistent. Recently, the relative abundances of fungal genera were evaluated using pyrosequencing approach in outdoor air samples from an urban US area and estimated relative abundance of fungi recognised to be allergenic from 2.8% to 10.7% of total fungal taxa [75]. Several studies did not find associations of outdoor fungal spore counts and visits to emergency departments or hospital admissions due to asthma exacerbations in adults [76,77], although both studies did find these associations in children. Fungal sensitisation was not investigated in these studies. In contrast, some studies have shown that sensitization to fungi *per se* is a risk factor for admission to an intensive care unit with an acute attack of asthma [78] and respiratory arrest [79]. Subjects who had been admitted to the ICU with severe life-threatening asthma were significantly more likely to have one or more positive skin tests for fungal dry weather spores (*Alternaria alternaria, Cladosporium cladosporoides, Helminthosporium maydis, Epicoccum nigrum*). O’Halloren et al. found that subjects who had a respiratory arrest during the *Alternaria* aeroallergen season were more likely to have a positive skin test to *Alternaria* than other subjects with asthma [79]. Delfino and coworkers found associations between fungal spore concentrations, particularly *Alternaria* and *Helminthosporium*, and increased asthma symptom scores, decreased evening PEF and β-agonist inhaler use in adult asthmatics sensitized to fungi [80]. Additionally sensitisation to *A. alternata* in asthmatic subjects with grass pollen sensitivity was found to predict susceptibility to thunderstorm-associated asthma [81]. Children living in rural environments are less likely to have asthma and fungal sensitisation [82], whereas adults are more likely to have ABPA, at least in India (47% vs. 66%, p = 0.007), which may or may not reflect airborne exposure [83]. It is likely however that these differences are not simply about indoor or outdoor air exposures, as the mould exposures in indoor air were higher in rural environments (in France) [84], and non-allergic mechanisms of asthma exacerbation were suggested.

##### Indoor

Moulds are commonly present in houses; a study evaluating the presence of different mould species in more than 1000 houses in Northeast USA revealed that *Cladosporium* spp is found in 85%, *Penicillium*, *Aspergillus* and *Alternaria* respectively in 75%, 50% and 28% of homes [85]. Reported mould exposure and recent water damage is associated with asthma symptoms and bronchial hyperresponsiveness in adult asthmatics [86]. This observed effect was consistent among 38 study centers in Europe and more prominent in subjects sensitized to *Cladosporium*. Sensitization to moulds is more prevalent in subjects leaving in damp dwellings compared to subjects living in dwellings without any sign of building dampness (9.3% vs 3.9% [87]). Furthermore, living in damp dwellings *per se* is associated with current asthma, lower FEV1 [88] and higher PEF variability [87]. Subjects sensitized to moulds, particularly to *Cladosporium* and *Alternaria* are at significantly higher risk of current asthma if they reported mould exposure [86,87,89]. Prevalence of current symptomatic asthma and use of asthma medications increase significantly with higher *Alternaria alternata* allergen levels indoor [90]. One study has shown that severity of asthma correlates significantly with measures of total dampness and mould growth in the dwelling [91]. However, numerous other studies were not able to confirm these findings. Wood and coworkers [92] did not find association between indoor mould and positive skin tests for common moulds. Several studies have shown no association between visible indoor moulds or airborne moulds and bronchial hyperresponsiveness [93,94].

### Epidemiology research priorities

• *Detailed and population based studies of the prevalence and annual incidence of SAFS, fungal sensitisation and ABPA/M, in adults and children.*

• *Improved and more geographically diverse exposure studies relating the nature of exposures to asthma and ABPA severity.*

There is accumulating evidence that mould exposure is associated with asthma, however no clear exposure-effect relations have been established. The only population-based incident case–control study providing such evidence has shown increased risk of asthma in relation to sIgE antibodies to *A. fumigatus* and *C. herbarum*[95]. Interestingly, the majority of studies do not provide evidence that increased prevalence of asthma associated with often assumed exposure to moulds is caused by the hypersensitivity to fungi. Furthermore, no consistent associations were found for the objective measures of asthma severity and control. This is in part because of inconsistency and inadequate validation of the measures used to evaluate exposure and health effects. Therefore, it currently remains impossible to set evidence-based guidelines for avoidance of fungi. Perhaps colonization or infection of the airways in affected people overshadows external fungal exposure, although a recent study found *A. fumigatus* isolation from sputum to be associated with elevated airborne levels in homes of patients with asthma [96].

### Pathogenesis

The pathogenesis of ABPA has been extensively studied, unlike fungal sensitisation and associated asthma. Although there is a correlation between the degree of exposure to *Aspergillus* spores and the development of ABPA, many findings suggest that ABPA is primarily the result of abnormal host immune response to *Aspergillus* antigens. Given that high incidence rates of ABPA have been reported in a number of families, such an abnormal host response is probably influenced by genetic factors [97,98]. While factors that allow growth of *Aspergillus* in the airways of ABPA patients remain unclear, bronchial mucus abnormalities have been implicated. For example, for unknown reasons, the mucus in ABPA can become extremely viscid and difficult to remove [99]. Clearly, complex interactions between host susceptibility and fungal development in the local pulmonary environment occur and need to be deciphered.

#### Lung microbiota

The application of sequence-based identification and metagenomics to studies of the human microbiome has revealed a plethora of organisms that can inhabit the human body including new or as yet unknown pathogens [100-102]. Most published studies have focused on bacterial communities, but fungi also form an important component of the microbiome [103]. The internal transcribed spacer regions (ITS1 and ITS2) of the nuclear ribosomal operon are the most popular loci used to identify and discriminate between fungal species and reference databases have been developed to allow comparison and identification of amplicons [104,105]. These regions have been the target of the few published lung fungal microbiota studies [104,106,107]. Unfortunately none have addressed ABPA, although a massive number of fungal species have been found in pooled sputum samples from asthmatics [108]. One area of uncertainty is the pathology of fungal growth within the lungs. Although in florid cases hyphae can be seen in mucus and there are old descriptions of bronchocentric granulomatosis (a distinctive immunopathology of allergic fungal disease), the extent and location of fungi in ABPA and SAFS affected lungs have not been well described.

Whilst fungal components do not need to be viable to elicit an allergic reaction, infection can only be caused by viable fungal cells. Conventional DNA-based methods for microbial identification are unable to differentiate between viable and nonviable cells, often resulting in an overestimation of microbial targets, which could be a major limitation. One approach that may circumvent this is viable-PCR (v-PCR), which utilises propidium monoazide (PMA) to detect only viable cells [109]. V-PCR has been applied to a number of microorganisms, including fungi, and has been used in conjunction with a number of DNA-based techniques including next generation sequencing. In sputum samples from individuals with cystic fibrosis, comparison of PMA treated and untreated clinical samples indicated that dead bacterial cells significantly bias untreated profiles [110].

Although high-throughput technologies have facilitated the identification and characterization of the human microbiome at mucosal sites including lungs, the mechanisms by which the commensal flora might influence lung immunity and their role in the development of allergic inflammation are not well characterized. Lung microbiota analysis is a new translational research area, offering the potential to redefine the processes that drives the progression of respiratory disease in asthma. Such studies will clearly impact our patient management, changing current paradigms of allergy/asthma and therapy.

#### Host-pathogen interactions

##### Neglect or react?

Every day we breath 20,000 times, thereby inhaling ~10,000 liters of air, which during the fungal season can often contain >50,000 fungal spores per cubic meter of air per day [111]. The size of these spores (approximately 2-50 μm) enables the smallest to reach the distal airways. For immune homeostasis it is critical to prevent excess inflammation and tissue damage in response to harmless species [112,113]. In response to the inhaled fungi, the pulmonary immune system has to decide whether to:

(i) tolerate the presence of these fungi or

(ii) to initiate an anti-fungal host defense program.

This decision and the resulting downstream signaling are critical for the integrity and maintenance of pulmonary host defense. These immunological ‘neglect’ or ‘react’ checkpoints are of key relevance for inhaled opportunistic fungi, in particular the filamentous saprophytic *A. fumigatus* or commensals, such as *Candida* species. These fungi can both colonize and infect the airways not only upon systemic immunosuppression, but also in pulmonary diseases where the local mucosal host defense is compromised, such as allergic asthma and cystic fibrosis [4,114-117]. Fungal colonization is the necessary antecedent event for sensitisation in the pathogenesis of ABPA, but it is unclear why only some individuals become sensitised. The factors determining the timing of sensitisation are not known and likely relate to a concurrent viral exposure and/or genetic predisposition (see Figure 2).

##### React

The pulmonary immune barrier function is mainly achieved by the lining epithelial cells, alveolar macrophages and pulmonary dendritic cells (DCs). These innate immune cells, are the first sentinels to recognize and react to inhaled fungi. Upon contact with fungi, airway epithelial cells produce large amounts of cytokines and chemokines, such as the prototypic interleukin 8 (CXCL8), which recruit leukocytes to the pulmonary site of infection to enhance antifungal host defense activities. The outcome of this initial interaction orchestrates the subsequent immune response. Macrophages phagocytose fungi, neutrophils phagocytose and/or release their granule contents and form DNA neutrophil extracellular traps (NETs) to entangle, immobilize and kill the fungus, a process involving neutrophil-derived calprotectin [118,119]. Even NK cells were recently found to potently fight *Aspergillus fumigatus*[120]. However, some fungi are capable of evading these host defense mechanisms, for instance *Candida*, by affecting phagosome maturation and phagosomal escape by formation of hyphae that destroy the vesicle and subsequently the entire host cell [121,122]. Neutrophils play the prime role in controlling *A. fumigatus* invasion and are essential in restraining tissue invasion and fungal spreading from the airways into the bloodstream [123,124]. In case of the allergic airway response to *Aspergillus*, the TH_2_-associated chemokine CCL17/TARC plays a key role in both murine and human fungal asthma [125,126]. CCL17 binds to CCR4 and thereby recruits TH_2_ cells into the airways that, in turn, drive IgE production and mast cell degranulation and feed the pro-allergic/-asthmatic airway response. Furthermore, CCL17 has been shown to impair macrophage killing of *Aspergillus*[125]. Since CCR4 is also expressed on Treg populations, these immunosuppressive cells may additionally favor survival of the fungus and a shift towards a TH_2_ response. Clinically, CCL17 may serve as a serum biomarker in patients to differentiate sensitization to *Aspergillus* from clinically active ABPA flares, especially when quantified longitudinally, as supported by two studies [127,128]; cutoffs are not established. Inspired by findings in animal models of ABPA, anti-CCL17 antibodies are currently being developed (Hogaboam CM, *personal communication*).

Among cells of adaptive immune system, particularly IL-17-secreting T-helper cells (Th17) play a key role in anti-fungal host defense [129,130]. Autosomal recessive IL-17RA and autosomal dominant IL-17F deficiencies have been reported in chronic mucocutaneous candidiasis (CMC) patients [131]. A recent study further shows that IL-17A itself is able to bind fungal cells, thereby causing nutrient starvation conditions [132]. The Th17 response works in concert with IL-22, IL-23 and Th1/Tregs responses to control fungal dissemination [133]. On the other hand, dysregulation of these pathways can drive exaggerated inflammatory responses/hyperinflammation as demonstrated in chronic granulomatous disease (CGD) in mice [134]. These studies also show that the indoleamine 2,3-dioxygenase (IDO), a key *enzyme* of tryptophan metabolism, is involved in CGD-associated inflammation and tryptophan catabolites (kynurenines) could be therapeutically useful to regulate and dampen hyperinflammation [135,136] although the regulation of this pathway in human CGD is different [137-140].

##### How are fungi recognized?

Similar to other microbes, fungi are recognized through conserved pattern recognition receptors (PRRs) of the innate immune system. Two families of PRRs are essential in fungal recognition:

• C-type lectin receptors (CLRs) and

• Toll-like receptors (TLRs) [118].

Prototypic CLRs include dectin-1, complement receptors, and macrophage mannose receptor. Less well-described include DC-SIGN, dectin-2 (recognizing house-dust mite), DCL-1, Mincle and CLEC5a. These CLR receptors have in common that they are coupled to Syk, which enables signaling via CARD9 and subsequent NFκB downstream activation [141]. Activation of these receptors by whole fungi through a proposed ‘phagocytic synapse’ [142] or fungal components (prototypically ß-glucans) elicit anti-fungal effector activities, that are (opsono)phagocytosis, cytokine production and respiratory burst. A recent study highlights that the interaction of *Aspergillus* conidia with the key CLR dectin-1 in the lung mainly takes places intracellularly in acidified phagolysosomes, following the phagocytosis of fungi by leukocytes [143]. The pathophysiological and clinical disease relevance of these receptors is corroborated by the finding that genetic mutations in distinct CLRs, namely dectin-1 [144] and CARD9 [145], cause *CMC* and other fungal infections. Beyond these pathways, STAT1 mutations have also been associated with CMC [146]. While genetic inheritance for susceptibility to *Candida* has been established, the molecular genetic basis for *Aspergillus infections in humans remains to be defined.*

In addition to CLRs, TLRs also recognize fungal patterns. The MyD88-coupled TLRs TLR1, TLR2, TLR4, TLR6 and TLR9 have been reported to contribute to fungal recognition in different experimental systems, with TLR2, TLR4 and TLR6 probably being the most important ones [147-150]. Cellular studies are supported by genetic association studies showing that one TLR4 polymorphism is weakly linked to susceptibility for invasive aspergillosis following haematopoetic stem-cell transplantation [151,152]. Studies with genetic knock-out mice mainly support the role of TLR2 and TLR4 in fungal recognition, but the specific receptor utilized depends on the phenotype of the fungus (stage-specific recognition, conidial *vs*. hyphal) and on the host genetic background (Brown GD, *personal communication*).

Besides cellular receptors, fungal patterns are recognized both by soluble innate immune receptors. Collectins, such as surfactant protein A (SP-A), SP-D, galectin-3, pentraxin-3, mannose binding lectin (MBL), C-reactive protein, complement, ficolins and others are *in vivo* the first proteins to bind fungi and facilitate the uptake and clearance by surrounding phagocytes. A recent study shows that opsonisation with surfactant components in the lung limits inflammation by attenuating host-fungi interactions, a mechanism relevant for zymosan but not for *Aspergillus* species [143], underscoring the complexity of host-fungus interactions in the lung.

### Pathogenesis research priorities

• *Define the lower respiratory tract microbiome in ABPA, SAFS, ABPM and in asthmatics sensitised to different fungi, combined with v-PCR and other measures of microbial viability.*

• *Study specific fungal factors and host components that are worth targeting in patients, in order to (i) improve clearance of the pathogen and to (ii) limit hyperinflammation-mediated tissue damage.*

• *The mechanisms of airway and lung damage in ABPA and SAFS. The impact of treatments, including corticosteroids, on those mechanisms.*

• *Studies of the immunological response to fungi to determine the relationship between TH*_*2*_*allergic responses and Th1/Th17 infection related responses.*

### Genetic associations

Several small studies have identified certain genetic factors linking the risk of ABPA with individual mutations. Multiple associations are to be expected given a family history in 5% of those with ABPA [98]. The genes implicated to date include IL-4Rα [153,154], IL-10 [155], surfactant A2 (SPA2) [156], TLR9 [30], CFTR [157-159], and HLA DR2/DR5 polymorphisms [160]. Some of these associations are not strong, and some may be related to disease progression or complications, rather than being risk alleles *per se*. All studies to date are relatively small. No genetic associations have been described with SAFS or other fungal allergic conditions.

### Genetic association/risk research priorities

• *Expand confirm and evaluate the number of genetic associations with ABPA and other fungal allergic conditions, compared with asthmatic patients without these fungal disorders.*

• *Define genetic pathways that regulate the susceptibility towards and damage response pathways in pulmonary fungal infection. Genetic and functional studies comparing patients with and without complications of ABPA and SAFS, including those with mild and severe bronchiectasis and those with early and advanced CPA*.

• *Risk associations in patients with and without disease could be direct associations with disease or indirectly related to complications such as bronchiectasis or worse pulmonary function. Thus association studies should include well documented complication rates and functional status over time.*

### Diagnosis and screening

A definitive diagnosis of the following 8 features to be present as follows: asthma; immediate *Aspergillus* skin prick test positivity (sensitivity and specificity: 94.7%, 79.7%); IgE levels >1,000 IU/mL (97.1%, 37.7%); positive *A. fumigatus* specific IgE (no value specified or studied) (100%, 69.3%); *Aspergillus* precipitins detectable (42.7%, 97.1%); eosinophil count >1,000 cells/uL (29.5%, 93.1%); transient or fixed chest radiographic opacities (36.1%, 92.5%); (central) bronchiectasis (91.9%, 80.9%); and high-attenuation mucus (39.7%, 100%) [5] (Table 2). So a key question is how these criteria should be applied in the routine clinical setting. Which are most useful as screening criteria to identify all those with ABPA? Further, can the same screening test(s) be used to identify patients with SAFS, also deserving of a trial of antifungal therapy? These questions have recently been addressed by an International Society for Human and Animal Mycology (ISHAM) working group report which proposed screening with a blood Aspergillus specific IgE assay, and a simpler definition of ABPA (see Table 1) [5]. Overall, no single test has both good sensitivity and specificity; hence multiple tests should be utilized for confirmation of ABPA. The diagnosis algorithm proposed by the ISHAM Working Group is to perform an *Aspergillus* skin test and/or *A. fumigatus* specific IgE levels (the latter one being more sensitive) and if either is positive other tests for ABPA (CT scan, IgG specific to *A. fumigatus*, serum precipitins to *A. fumigatus* and total eosinophil count) should then be performed to establish the diagnosis of ABPA. What arguments are in favor of this approach, or another? Performance variability in the screening tests used to identify ABPA, SAFS, and those sensitised to fungi are pivotal to diagnosis, estimates of prevalence and understanding pathogenesis.

**Table 2:** Overview of screening tests applied to diagnosis of fungi-associated conditions

**Test**	**Strengths**	**Weaknesses**	**Comment**
Skin prick test (SPT)	Simple to perform. Rapid results. Good tolerability. Inexpensive. High negative predictive value (95%)	Accuracy and reliability dependent on quality of fungal extracts. Variability between different batches of test.	Test should be performed with standardised allergen solution, if possible. Best used in conjunction with sIGE due to discordance in results.
		Misses low sensitivity responses.	
		Systemic and topical antihistamines may suppress weal and flare reaction. Presence of IgE without clinical symptoms.	
Intradermal tests	More sensitive than SPT.	Higher rate of false positives than SPT.	Rarely used.
IgE (ImmunoCAP)^a^	Completely safe. Not influenced by concurrent drug treatment.	Results not immediately available. Testing more expensive than SPT. Presence of IgE without clinical symptoms.	Best used in conjunction with SPT due to discordance in results.
Complete blood count	Automated test.	Many different conditions result in increases or decreases in cell populations. Lack of correlation between peripheral blood eosinophil levels and lung function / immunological parameters.	Performed to assess peripheral blood eosinophil levels.
Fungal culture of sputum	Simple to perform. Inexpensive.	Lack of standardisation in most countries. UK standard insensitive.	Actively growing culture needed for strain-typing or anti-fungal sensitivity
Fungal PCR of sputum	More sensitive than culture.	May have high false-positives. Requires specialised equipment, although most labs have PCR machines.	Commercial tests available but no accepted standard for positivity.

^a^ = often referred to as RAST tests, although the original radioallergoabsorbent test has been superceded by the ImmunoCAP or simpler ELISA tests.

The skin-prick test (SPT) is a simple diagnostic tool that can be useful for screening for ABPA [161], but it is not without limitations. The accuracy and reliability of the SPT and other *in vivo* and *in vitro* assays is highly dependent on the quality of the fungal extracts used. The quality can vary dramatically between commercial suppliers, which can be caused by inconsistencies in the preparation of fungal extracts; for example, using either fungal mycelia or spores for production. Inter-strain variability leads to extracts with altered protein composition, resulting in poorly standardised allergy testing solutions [162]. The majority of SPT positive individuals are also positive by specific IgE, giving the SPT a high negative predictive value (95%); however a significant proportion of individuals with positive IgE tests are SPT negative [4]. Intradermal tests are more sensitive than SPT [163]; however, these are rarely performed and carry a higher rate of false positives. *In vitro* measurement of multiple specific IgE antibodies may be more costly than SPTs. The CAP system for specific serum IgE testing has higher sensitivity than RAST tests with comparable specificity; and a recent study has suggested that both SPTs and specific IgE measurement by the (ImmunoCAP) system should be used in diagnoses of fungal allergy, due to discordance in test results in around a quarter of severe asthma patients [43]. Specific recombinant allergen testing may play a greater role in diagnosing ABPA in the future. Specific *A. fumigatus* allergens have been evaluated for their diagnostic performance in serologic studies in asthmatic patients [164], and data suggests they may be useful in discriminating between ABPA (Asp f 2, Asp f 4 and Asp f 6) and fungal allergy (Asp f 1 and Asp f 3) [165,166]. Aspergillus precipitating antibody is found in <50% of ABPA patients and fewer SAFS patients, and is therefore a poor screening tool. Likewise radiology is insensitive and non-specific enough in ABPA and wholly unsatisfactory for screening for SAFS, in which radiology is often normal or nearly so.

Classic ABPA cases in the absence of oral corticosteroids have peripheral blood eosinophilia [167]; however, this feature may be only present at the time of exacerbation or during the acute phase of the disease [168]. A peripheral blood eosinophil count of above 1,000 cells/ul is often cited as a secondary diagnostic criterion and whilst it is still considered to be suggestive of ABPA [4] the utility of it as a diagnostic tool has been questioned [20,168], as most patients with ABPA have fewer eosinophils than this on presentation. One study to address this found no correlation between peripheral blood eosinophil levels and lung function or other immunological parameters; and whilst prevalence of central bronchiectasis was higher in patients with higher eosinophil counts, the severity of bronchiectasis did not correlate with the degree of peripheral eosinophilia [20]. Consensus of 500 cells/ul (at presentation or previously) was reached by the ISHAM working group, and needs prospective validation.

Culture of *A. fumigatus* from sputum is supportive but not diagnostic of ABPA, as the fungus can also be cultured from patients with other pulmonary diseases [169]. Sputum samples from individuals with asthma are usually culture negative [24]. The standard approach used to process sputum for mycological investigations in the UK has been shown to potentially underestimate fungal prevalence in the airway [170], and a recent multi-centre study has highlighted the variability in culture results that arise from different mycological methods being employed [171]. As such a number of groups have questioned the performance of the current UK respiratory culture methods for fungi and have called out for an improved, standardised approach [170-173]. PCR has been proposed as an alternative or additional approach to detecting *Aspergillus* species in sputum and has been shown to be more sensitive than culture in ABPA but needs to be interpreted with other clinical and laboratory features [174]. DNA-derived signals can originate from nonviable fungal cells [175,176] and therefore there is the potential for a positive PCR signal to derive from dead fungal material, particularly in the context of a patient who has been treated with antifungals.

### Diagnosis and screening research priorities

• *Develop more standardized skin test and blood test reagents for detecting fungal sensitization, with validation in different populations with other potential causes of elevated IgE, such as chronic helminth infection.*

• *Assess the performance of better standardized tests for detecting ABPA and SAFS in asthmatics seen in routine clinical practice.*

• *Compare the performance of simpler combinations of tests to diagnose ABPA and SAFS, not including complications as a component of the diagnostic definition.*

To summarise, the current tests used to determine fungal sensitisation are not without limitations, and the discordance between test results must be taken into consideration. The use of specific recombinant allergens to discriminate between ABPA and fungal allergy has shown great potential and may be of importance in the future. The detection of *A. fumigatus* and other fungi from clinical specimens such as sputum is highly dependent upon the methodology used, and a more sensitive approach that is standardised and universally adopted is a definite need.

### Clinical management

There are 5 aspects to the management of fungal allergy in asthma. These are:

1. Avoidance of fungi

2. Control of the inflammatory process

3. Improvement of airway air flow through reduction of mucus and obstruction

4. Reduction of fungal burden

5. Control of bacterial infection

#### Decreasing indoor exposure to moulds

Allergen avoidance remains the cornerstone in the management of any allergic condition. Several interventional studies employing remediation of damp buildings [177], removing visible mould [178], removing water damaged materials [179-181], applying fungicides [178], making alterations to heating [182], improving ventilation [178] and fixing buildings construction to prevent further water leakage [183] have been conducted. The interventions were conducted either at home [178,182,183] or in the working place [179-181,184,185].

Studies performed in occupational settings have shown that employees more commonly report improvement of symptoms while being away from work [179] and indicated a reduction of respiratory symptoms including cough, shortness of breath, wheezing [185], stuffy or runny nose, eye, throat, skin, and headache symptoms [179] after evacuation/relocation from the infected building [179,185]. In active intervention studies patients in remediation groups reported significant reduction of bronchitis [180], improvement in wheeze affected activities, perceived reduction in asthma medications (both relievers and controllers), symptoms of rhinitis [178] including allergic rhinitis [180], conjunctivitis [180,184], rhinoconjunctivitis [178], complaints on irritated eyes or nose and skin rash [181]. When remediation is compared to no remediation, a significant decrease in reported wheezing [178,183], need for asthma relievers and controllers [178] and trend toward fewer hospital admissions for respiratory conditions [183] in those patients whose homes or work places had remediation undertaken. However, other studies failed to show a beneficial effect of intervention [182,184,186]. Inconsistent effects of remediation on lung function are also noted [178,180,181].

Farm environments could represent an occupational risk, as reported from India [83]. Specifically poultry farms may represent a local major source of fungi [187,188] and poultry workers have a higher rate of asthma [189,190] and mould IgG antibodies [189]. Protection of these workers may be important, but is not studied.

Numerous caveats accompany these interventional studies and they have several limitations. Interventions are applied indirectly to a particular participant as it the building is usually that receives attention. Moreover, usually it was impossible to determine a separate effect of mould reduction from interventions themselves such as increasing indoor temperature toward normal level could influence health. Some ‘common sense’ avoidance measures are usually recommended to affected patients such as avoidance of all composting, changing pillows regularly, avoiding building works, especially of old buildings, cellars and loft spaces. The evidence for these avoidance measures is limited [191]. Currently, additional studies are required to establish evidence-based guidelines on mould avoidance.

#### Control of the inflammatory process

Inhaled corticosteroids have transformed the management of asthma. Regular use in symptomatic asthmatics reduces mortality, but their impact in those with minimal or intermittent symptoms is not clear and they may be detrimental. One small prospective multicentre randomized controlled trial (RCT) in ABPA comparing beclomethasone and placebo showed no overt benefit and an increase in the frequency of *Aspergillus* isolation from sputum in the active group [192]. Despite beclomethasone, seven patients had clinical exacerbations as well as more radiological exacerbations than placebo. A 23 patient study of ABPA patients without bronchiectasis treated with inhaled corticosteroids showed subjective improvement but without complete control of asthma and median IgE levels increased [193]. The impact of inhaled steroids on ABPA (as opposed to asthma) is unclear and may only be necessary to improve asthma control. Inhaled corticosteroids are insufficient alone in controlling ABPA or fungal colonization, and could be detrimental in the latter, but this is unstudied.

There are no RCTs trials of systemic corticosteroids in ABPA although they are generally accepted as appropriate therapy for acute exacerbations. However few data are available to guide either dose or duration of corticosteroids. Lower doses of corticosteroids without antifungal therapy (0.5 mg/kg/day for 1-2 weeks, then on alternate days for 6-8 weeks then taper and discontinue) is associated with 45% more relapse and subsequent oral corticosteroid dependence [167]. A higher dosage of corticosteroid for longer (0.75 mg/kg for 6 weeks, 0.5 mg/kg for 6 weeks, then tapered by 5 mg every 6 weeks to continue for a total at least 6 weeks) was associated with a higher remission rate and a lower prevalence of corticosteroid-dependent ABPA (13.5%) [57]. Parenteral steroids may be required for ABPA unresponsive to oral steroids and antifungals, and may down-regulate steroid receptors [194]. Whether the benefit of short-term control with oral corticosteroids correlates with longer-term outcome has not been assessed. A RCT on the efficacy and safety of two different glucocorticoid dose regimens in ABPA (clinical trials.gov; NCT00974766) has been completed and results awaited.

Omalizumab has an established place in the management of some patients with severe asthma. Omalizumab reduces symptoms, exacerbations, asthma hospitalizations and has a steroid-sparing effect. While a recent study indicates that omalizumab is associated with improvements in outcomes in patients with uncontrolled persistent allergic asthma [195], the very high IgE in ABPA makes the use of omaluzimab difficult because of cost and administration limitations (volume). Whether those patients with SAFS should be first trialed on antifungal therapy or omaluzimab or given both is not known.

Macrolide therapy (especially azithromycin) has an anti-inflammatory role in some patients to decrease cough and sputum production. This unexpected role was discovered following the improvements noticed in patients with diffuse panbronchiolitis mainly found in people from East Asia [196]. It has been used in those with bronchiectasis, especially those with frequent exacerbations [197], as well as for other pulmonary diseases [198]. It has not been studied in ABPA and in particular the relative efficacy of macrolide versus itraconazole, in patients with and without bronchiectasis is lacking.

#### Improvement of airway air flow through reduction of mucus and obstruction

Nebulized hypertonic saline (6-7%, 4-5 mL) can be used to reduce the viscosity of sputum to ease expectoration of mucus plugs encountered in ABPA [199]. As hypertonic saline can also induce bronchoconstriction [200], caution is required with its initial administration especially in those with a low FEV_1._ Its role in ABPA has not been actively addressed.

Mucus plugging of the airway is a characteristic feature of ABPA. Mucus is usually hypoattenuated, but may be hyperattenuated on CT scan. The removal of mucus plugs will generally improve clinical status and lung function. Indeed mucoid impaction is one uncommon presentation of ABPA. A course of oral steroids can sometimes relieve obstruction. The role of either nebulised hypertonic saline or N-acetyl cysteine in contributing to mucus plug removal is not studied. Therapeutic bronchoscopy (flexible or rigid) is often used but its efficacy and medium term impact is not studied or described. Hyperattenuated mucus plugging may be more difficult to relieve and is prone to relapse [58], and the most effective means of removal of hyperattenuated mucus and preventing relapse is not known.

#### Reduction of the fungal burden

Ketoconazole and inhaled natamycin were unsuccessful therapies for ABPA and have not been attempted for SAFS. There are no published data about nebulised amphotericin B for either ABPA or SAFS. Itraconazole was the first orally active agent against *Aspergillus* species and many other filamentous fungi. Two randomized placebo-controlled studies which enrolled a total of 84 patients both demonstrated benefit compared with placebo [201,202]. However the outcome parameters used in the studies were different and were composite in the first study. Pooled analysis showed that itraconazole reduced IgE levels by >25% when compared to placebo but did not significantly improve lung function. In the earlier, slightly larger study, the overall response rate was 60%, with a number needed to treat (NNT) of 3.58, consistent with a very effective intervention. Patients with ABPA may have continuing symptoms (asthma, bronchiectasis) as well as acute exacerbations. These were not separated out in terms of responses. Our own extensive experience is consistent with a range of responses, from dramatic through minor improvement to a complete lack of effect; the reasons for this response heterogeneity, despite adequate drug concentrations in blood, is unclear. Relapse after conclusion of active therapy was not reported in either study. The frequency of acute exacerbations is not well known in ABPA, although was infrequent (16 of 41 (39%) over a mean period of 43 months) in a group of patients without bronchiectasis [193].

Fluconazole and itraconazole are suggested as possible effective treatments in the *Tricophyton* asthma [203], a phenotype of uncertain frequency. Fluconazole was effective in improving asthma in a small RCT in moderately severe asthmatics sensitized to *Trichophyton*, many with cutaneous fungal infection [204]. This has never been followed up. The use of terbinafine, which is active against *Trichophyton* spp., is only anecdotal [205]. The relative efficacy of fluconazole in those with multiple fungal sensitisation is not known, and is predicted to be inferior to itraconazole as it has a much more narrow spectrum of activity.

A broader approach was subsequently trialled using the wider spectrum agent itraconazole in SAFS patients sensitized to one or more of 7 fungi [27]. Compared to placebo, those treated with itraconazole over 8 months had a significant benefit in the primary endpoint of quality of life, as well as a fall in total IgE and marginal improvements in morning peak expiratory flow rate. The study was small (58 patients) and the confidence intervals just overlapped, so a replication study is warranted. Other data suggested a similar effect, but with some different parameters of response including reduced peripheral eosinophilia and improved FEV1 [206]. As with ABPA, our clinical experience suggestes some dramatic responses and some failures, with no clear cut reason discerbable for the variable response. While the effect size was similar to that seen in ABPA (NNT 3.22), the place of itraconazole in the management of severe asthma is not yet well defined.

The newer triazoles voriconazole and posaconazole have been effective in some patients with ABPA and SAFS, primarily in cystic fibrosis, but latterly in asthma [29]. Most experience to date has been in those intolerant to itraconazole or in itraconazole failures. Two remarkable findings in this retrospective review however were: (1) the large numbers of patients who were able to come off oral corticosteroids and substantially reduce their corticosteroid burden, and (2) that a third of the patients had their asthma severity downgraded from severe to moderate. These findings suggest a powerful impact in a proportion of ABPA and SAFS patients, which requires additional study (such as ATCF study, which is underway and aims to evaluate the rates of converting sputum cultures for *Aspergillus* from positive to negative, for itraconazole and voriconazole in a large prospective controlled clinical trial of CF patients). Unfortunately, the recently published EVITA3 study failed to show improvement in quality of life or the number of severe exacerbations during 9 months after termination of a 3 months’ course of voriconazole in refractory asthma associated with sensitization to *A. fumigatus*[207]. Voriconazole 200 mg bd was given for only three months and while there was a significant reduction in the degree of *A. fumigatus* colonization in the voriconazole group the drug did not eradicate the mould from the sputum during the treatment period and rates of positive culture returned to baseline within a few months of stopping treatment. The placebo group had a surprisingly good improvement rate, and quality of life is often adversely affected by voriconazole, because of side effects.

As the bioavailability of itraconazole is variable and inadequate exposure is associated with higher clinical failure rates, therapeutic monitoring is required and is often not done or available. Variable absorption from different formulations of itraconazole contributes to the difficulties of maintaining consistent exposure and therefore effect. The penetration of itraconazole, voriconazole and posaconazole into sputum and extracellular lung fluid (ELF) is variable and often low, despite adequate serum concentrations, for unclear reasons [208]. Voriconazole administered IV yielded high mean ELF concentrations (range 10.1-48.3 mg/L) and a high penetration ratio (7.1). In contrast, mean ELF concentrations of oral itraconazole and posaconazole were 0.2-1.9 mg/L, and the ELF penetration ratios were <1 [209]. However both itraconazole and posaconazole are concentrated inside alveolar cells.

The optimum usage of itraconazole and other oral azoles for therapy in ABPA is unclear presently, despite the 2 RCTs described above. Monotherapy of itraconazole in ABPA is possible but not studied. A randomized controlled trial comparing monotherapy of itraconazole versus prednisolone in ABPA (MIPA study; clinical trials.gov; NCT01321827) is underway, which aims to answer this question. The duration of therapy and frequency of relapse is unclear in ABPA, partly because exacerbation rates are infrequent. Reversion to pre-therapy status within 4 months was almost universal in SAFS patients who discontinued itraconazole therapy [27]. A recent study examining the impact of itraconazole on Vitamin D receptor expression in CF patients suggests a prolonged effect of down-regulation, lasting significantly longer than itraconazole therapy, possibly indicating that after successful therapy a sustained effect may be found in some patients [210].

The negative effects of itraconazole and possibly other azoles, other than their own direct toxicities, include a marked reduction in inhaled corticosteroid metabolism (with corresponding boosting of exposure) and azole resistance in *A. fumigatus*[211,212]. In a UK study, profound boosting of systemic corticosteroid exposure with inhaled corticosteroids was seen in 50% of patients treated with itraconazole, which reversed after 8 months [27]. Interactions between both budesonide and fluticasone and itraconazole are documented, and are likely with voriconazole and posaconazole, although may be less marked. Ciclosonide and beclometasone do not appear to interact with azoles. Adrenal failure is however described, typically after months or years of dual therapy.

#### Control of bacterial infection

A high frequency of respiratory bacterial infections is common in many of these patients. Most are presumed rather than documented, but bronchitis attributable to *Streptococcus pneumoniae* and *Haemophilus influenzae* is common, especially in those with bronchiectasis [213]. Long-term administration of azithromycin or doxycycline can alleviate some of these infections. Low specific antibodies to these bacteria are common in these patients and occasionally this is the presentation of hypogammaglobulinaemia. Immunisation with Pneumovax or Prevnar 13 to prevent *S. pneumoniae* infection and Menitorix to prevent *H. influenzae* is often done, but not well studied [214-217]. Individual immunisation responses are highly variable, and often poor to Pneumovax. Reduction of exacerbations and recurrent infection has a major benefit for patients, and reduces corticosteroid courses.

### Treatment and general management research priorities

• *Additional studies on avoidance strategies to prevent fungal sensitisation in established asthma and course/exacerbations of fungi-related asthma and conditions related to incidental fungal exposures.*

• *The mechanism of benefit of antifungal therapy needs clarification, with development of biomarkers for response assessment.*

• *RCTs of voriconazole, posaconazole, isavuconazole and nebulized amphotericin B need to be conducted in ABPA and SAFS.*

• *Studies on antifungal and anti-IgE treatments in SAFS*.

• *The role of adjunctive therapies such as macrolide, antibiotic prophylaxis, mucus reduction, the efficacy and impact of anti-Pseudomonas therapy needs to be assessed.*

• *The avoidance and management of azole/corticosteroid drug: drug interactions needs addressing.*

• *The dual interaction of antifungal development of azole resistance and/or acquisition of a resistant strain on therapeutic impact requires addressing, given the increasing frequency of azole resistance in A. fumigatus.*

• *The optimal means and impact of reducing amplification of inflammation, through prevention of bacterial and viral intercurrent infection.*

Some patients become infected and others colonised by *Pseudomonas aeruginosa*, usually those with bronchiectasis [218]. Strategies to eradicate this with intravenous, dual antibiotic therapy are commonly used, without any clinical trial basis. The standard registered doses of ciprofloxacin are inadequate for most patients [219,220] and high doses such as 500-750 mg three times daily are more efficient. Newer approaches include oral high dose ciprofloxacin combined with nebulized colistin, following the lead of the cystic fibrosis community [221]. Data are lacking on impact and efficacy.

## Competing interests

The authors declare that they have no competing interests.

## Authors’ contributions

All authors were involved in the Task Force discussions and contributed to writing the document. All authors read and approved the final manuscript.

## Task Force membership

David W. Denning, Catherine Pashley, Svetlana Sergejeva, Domink Hartl, Andrew Wardlaw, Cendrine Godet, Stefano Del Giacco, Laurence Delhaes, Antoine Magnan, Eckard Hamelmann, Jaime Sanchez, Joan Bartra, Peter Hellings, Juan Luis Rodriguez Tudela, Joaquim Mullol.
